# Effect of Tai Chi Synergy T1 Exercise on Autonomic Function, Metabolism, and Physical Fitness of Healthy Individuals

**DOI:** 10.1155/2018/6351938

**Published:** 2018-06-27

**Authors:** Hsu-Chih Tai, Yi-Sheng Chou, I-Shiang Tzeng, Cheng-Yu Wei, Chun-Hsien Su, Wen-Chen Liu, Woon-Man Kung

**Affiliations:** ^1^Department of Exercise and Health Promotion, College of Education, Chinese Culture University, Taipei, Taiwan; ^2^Institute of Clinical Medicine, School of Medicine, National Yang-Ming University, Taipei, Taiwan; ^3^Division of Hematology and Oncology, Department of Medicine, Taipei City Hospital, Renai Branch, Taipei, Taiwan; ^4^Department of Research, Taipei Tzu Chi Hospital, Buddhist Tzu Chi Medical Foundation, New Taipei City, Taiwan; ^5^Department of Neurology, Chang Bing Show Chwan Memorial Hospital, Changhua, Taiwan; ^6^Department of Leisure and Sports Management, College of Life and Creativity, Cheng Shiu University, Kaohsiung, Taiwan; ^7^Division of Neurosurgery, Department of Surgery, Taipei Tzu Chi Hospital, Buddhist Tzu Chi Medical Foundation, New Taipei City, Taiwan; ^8^Department of Surgery, School of Medicine, Buddhist Tzu Chi University, Hualien, Taiwan

## Abstract

**Objectives:**

Tai Chi synergy T1 exercise is an aerobic exercise derived mainly from Tai Chi exercise. It is also derived from the Eight Trigrams Palms, form and will boxing, mantis boxing, Qigong, and Yoga, with a total of 16 sessions in 63 minutes. In this study, we investigated its effects on autonomic modulation, metabolism, immunity, and physical function in healthy practitioners.

**Method:**

We recruited a total of 26 volunteers and 23 control participants. Heart rate variability (HRV), blood pressure, and body mass index (BMI) were recorded before and after practicing Tai Chi synergy T1 exercise and regular walking for 10 weeks, respectively. Serum glucose, cholesterol, and peripheral blood including B and T cell counts were also measured. They underwent one-minute bent-knee sit-ups, sit and reach test, and three-minute gradual step test.

**Results:**

Tai Chi synergy T1 exercise enhanced parasympathetic modulation and attenuated sympathetic nerve control with increased very low frequency (VLF) and high frequency (HF) but decreased low frequency (LF) compared to the control group. Metabolic profiles including serum glucose, cholesterol, and BMI significantly improved after exercise. The exercise enhanced innate and adaptive immunity by increasing the counts of CD3+ T cells, CD19+ B cells, and CD16+CD56+ NK cells but decreasing the CD3+ cytotoxic T cell count. All monitored parameters including physical fitness and physical strength improved after the exercise.

**Conclusion:**

Tai Chi synergy T1 exercise improves autonomic modulation, body metabolism, physical fitness, and physical strength after 10 weeks of practice.

## 1. Introduction

Tai Chi exercise, a form of Chinese shadow boxing, is one of the major branches of the traditional Chinese martial arts. It originated in ancient China and is used for self-protection and health promotion. The spirit of Tai Chi exercise focuses on the balance and coordination of breathing, body movements, and mental concentration. It is practiced widely in Taiwan with several sessions of continuous rhythmic movements involving dynamic shifts of body weight from one foot to the other and rotations similar to aerobic exercise [1]. It is beneficial for maintaining cardiovascular function. For example, Tai Chi exercise can reduce serum ET-1 and TG in elderly people [2].

Heart rate variability (HRV) is the fluctuation of the R-R interval (RRI) in electrocardiograms (ECGs). It contributes to the understanding of the complex autonomic control of cardiac rhythm and explains the autonomic modulation of individuals [3]. Frequency-domain analyses have revealed that HRV in the high-frequency region within the range of 0.15-0.4 Hz is generated by the parasympathetic activity, predominantly from the vagal nerve, while that in the low-frequency region within the range of 0.04-0.15 Hz is generated by the parasympathetic and sympathetic activity. It can also be applied to psychiatry [4]. Some studies have reported that, by harmonizing slow, deep breathing, motions, and mind, Tai Chi exercise can tilt the balance of autonomic control from sympathetic to parasympathetic dominance [5–7]. Estimation of power spectral density (PSD) is the method of calculating frequency-domain measures to quantify the relative power of different band frequencies. The total power (TP) and low frequency (LF) are components mediated by sympathetic and parasympathetic modulation. The very low frequency (VLF) is related to the thermoregulatory mechanism, fluctuation in the activity of the renin ± angiotensin system, and the function of peripheral chemoreceptors. The high frequency (HF) component reflects the parasympathetic activity. HF% represents sympathetic inhibition. LF% has been used as an indicator of sympathetic tone. The LF to HF ratio (LF/HF) resembles sympathovagal balance [8, 9].

Tai Chi synergy T1 exercise is an aerobic exercise composed of movements derived not only from Tai Chi exercise but also from Eight Trigrams Palms, form and will boxing, mantis boxing, Qigong, and Yoga and was developed by Hsu-Chih Tai at Chinese Culture University. The 60-minute exercise involves 4 exercise elements: handwork, trunk work, legwork, and whole-body work. The 3 levels of exercise intensity, light, average, and heavy, are adjusted according to the tolerance and fitness of the exerciser. Many aspects of exercise were incorporated, including muscle strength, muscular endurance, explosive force, flexibility, sense of balance, coordination, and impact exercise (e.g., hand clapping or foot tapping). The main postures of Tai Chi synergy T1 exercise consist of the horse stance, lunge, stand-alone stance, telemark step, crouching, body twist and wring, and standing stooped, face upward. There are 16 sessions, and the details of the physiology of Tai Chi synergy T1 exercise are summarized in Table 1.

Our aim in this study was to investigate the physiological effects of Tai Chi synergy T1 exercise, including HRV, blood pressure, body mass index (BMI), and bone density, the biochemical effects, including serum glucose and cholesterol, and the effects on physical functioning, including physical fitness, and on the immunity of healthy individuals.

## 2. Participants and Methods

### 2.1. Study Design, Participant Population, and Data Collection

The study was approved by the Institutional Review Board of Chinese Culture University with approval numbers 98014 and 98018. This community program of health promotion in Hsinchu City was conducted at Hsinchu Armed Forces Hospital. A total of 26 volunteers with a past history of medical illness or surgery were recruited for the study; they were 3 males and 23 females. The participants were required to sign an informed consent before enrollment. They practiced Tai Chi synergy T1 exercise for 60 minutes once every week for at least 2 hours after meals during a 12-week period.

A total of 26 participants were enrolled (3 males and 23 females) in this community-based study. Subjects were excluded if they had missing data for age, sex, anthropometric values, and blood test results. All presentations (i.e., Mean ± SD) in Table 1 were based on the complete dataset. Before enrollment, the participants had to document their past medical history, including cardiovascular or metabolic disease and history of medication use in the most recent 3 months by filling out questionnaires. Their body weight, height, proportions of body fat, and fat-free mass were measured using a weight-height scale (DS 102, Jenix) and body composition analyzer (InBody 3.0, Biospace), respectively. Both before and after 12 weeks of practicing Tai Chi synergy T1 exercise, the heart rate, systolic blood pressure, and diastolic blood pressure of the participants were recorded and analyzed for 5 minutes using Polar RS800 with the Windows XP (Microsoft) operating system. Intervals of heart rates were transformed to frequency domain by discrete Fast Fourier Transform (FFT) and expressed by power spectral density (PSD) or spectral distribution as 5-minute total power (TP), very low frequency (VLF), low frequency (LF), high frequency (HF), and ratio of LF/HF. The serum glucose and cholesterol of the participants were checked before and after exercise using an automatic biochemical analyzer (SPOTCHEM SP-4430). BMI was calculated using the following equation: body weight (kilograms) divided by body height^2^ (meters^2^). Physical fitness was measured by one-minute bent-knee sit-ups, the sit and reach test, and the 3-minute gradual step test. Bone density was measured by quantitative ultrasound scanning of calcaneus bone (Medilink/PEGASUS, France).

We provided a control placebo employing similar intensity or METs as Tai Chi synergy T1 exercise, with participants under moderate-intensity regular walking. Our regular walking control group with similar baseline characteristics performed walking exercise on level ground at 5-5.5 kilometers-per-hour for 60 minutes. This control has an intensity of approximately 3.5-4 METs, which is comparable to that of Tai Chi synergy T1 exercise. A total of 23 participants were enrolled as a control group in this community-based study. The participants also had to document their past medical history, including cardiovascular or metabolic disease and history of medication use in the most recent 3 months by filling out questionnaires before enrollment. Similar to the case group, their body weight, height, proportions of body fat, and fat-free mass were measured using a weight-height scale (DS 102, Jenix) and body composition analyzer (InBody 3.0, Biospace), respectively. Enrolled participants were instructed to walk at a suitable pace by the instructor in the first session. The intervention lasted for 12 weeks, which was consistent with that of Tai Chi synergy T1 exercise group. Both before and after 10 weeks of regular walking, the heart rate, systolic blood pressure, and diastolic blood pressure of the participants were recorded and analyzed for 5 minutes using Polar RS800 with the Windows XP (Microsoft) operating system. Intervals of heart rates were transformed to frequency domain by discrete Fast Fourier Transform (FFT) and expressed by power spectral density (PSD) or spectral distribution as 5-minute total power (TP), very low frequency (VLF), low frequency (LF), high frequency (HF), and the LF to HF ratio (LF/HF). Serum glucose and cholesterol were also measured. Participants also underwent physical fitness and strength evaluation including one-minute bent-knee sit-ups, sit and reach test, and three-minute gradual step test. However, the serum tests in the control group were not performed due to limited funding.

The cell counts of immune regulator cells, including T cells, CD3+ cells, CD19+ B cells, CD16-CD56- cytotoxic T cells, and CD16+CD56+ NK/T cells, were measured in the laboratory of the National Research Institute of Chinese Medicine, Ministry of Health and Welfare, by flow cytometry (BD FACSCalibur, USA).

### 2.2. Statistical Analysis

Outcome measurements before and after exercise, including HRV, blood pressure, BMI, serum glucose and cholesterol, bone density, and index of physical fitness, were compared by paired *t*-test or Wilcoxon's signed-rank test. A *p* value less than 0.05 in the analysis was determined as statistically significant in 2-sided tests. Statistics were analyzed using SPSS software (version 17.00, SPSS, Chicago, IL, USA).

## 3. Results

### 3.1. General Characteristics of Volunteers

A total of 26 volunteers (3 males and 23 females) with a median body height of 157 cm, body weight of 60.5 kg, and age of 45.4 years were enrolled. A total of 23 participants were enrolled as a control group in this community-based study with a median body height of 160 cm, body weight of 55.0 kg, and age of 45.0 years.

### 3.2. General Characteristics of Tai Chi Synergy T1 Exercise

There are 16 sessions in the Tai Chi synergy exercise program, and the total duration is 63 minutes (Table 1). Heart rates range from 87 to 130 beats per minute. The respiratory exchange ratio lies between 0.8 and 1.0, except that of the 14th and 16th sessions. The total energy consumption is 245 Kcal. The intensity of this exercise is approximately 3.69 (± 0.87) metabolic equivalent of tasks (METs). There are no serious adverse events reported in the participants related to practicing Tai Chi synergy T1 exercise.

### 3.3. Ten-Week Effect of Tai Chi Synergy and Regular Walking on HRV

Compared with baseline, the VLF [ln (ms2)], HF [ln (ms2)], LF [ln (ms2)], HF% [nu], and TP increased significantly after practicing Tai Chi synergy exercise, but the LF% [nu] decreased significantly. There was no significant difference in LF/HF [ln (ratio)] before and after practicing Tai Chi synergy exercise (Table 2). For the control group, all parameters of HRV had no significant difference before and after practicing regular walking (Table 2).

### 3.4. Ten-Week Effect of Tai Chi Synergy and Regular Walking on Blood Pressure, BMI, Glucose, Cholesterol, and Bone Density

Systolic, but not diastolic, blood pressure, BMI, serum glucose, and cholesterol all significantly decreased after Tai Chi synergy exercise. However, no significant difference was noted in bone density before and after exercise (Table 3). For the control group, blood pressure, glucose, cholesterol, and bone density show no significant difference before and after practicing regular walking. However, BMI had significant difference before and after practicing regular walking (Table 3).

### 3.5. Ten-Week Effect of Tai Chi Synergy and Regular Walking on Physical Fitness and Physical Strength

Participants had significant improvement in the index exercises of physical fitness, including one-minute bent-knee sit-ups, the sit and reach test, and the 3-minute gradual step test (Table 3). Participants in the control group had no significant improvement in the index exercises of physical fitness, including one-minute bent-knee sit-ups and the 3-minute gradual step test. The improvement in the sit and reach test was significant (Table 3).

### 3.6. Ten-Week Effect of Tai Chi Synergy on Immune Regulator Cells

After practicing Tai Chi synergy T1 exercise, the cell counts of T cells, CD3+ T cells, CD19+ B cells, and CD16+CD56+ NK cells all significantly increased; only CD3+ cytotoxic T cells decreased significantly (Table 4).

## 4. Discussion

In this study, we first demonstrated the details of 16 sessions of Tai Chi synergy T1 exercise, including the duration, respiratory exchange ratio, and energy consumption, with the total energy consumption being 245 Kcal when the entire course of 63 minutes was practiced. Second, by practicing Tai Chi synergy T1 exercise, the seesaw of autonomic activity was tilted from sympathetic tone (LF% decreased from 53.74 nu before exercise to 47.52 nu after exercise) to parasympathetic tone (HF% increased from 30.58 nu before exercise to 34.98 nu after exercise) and the ratio of LF/HF, indicating sympathetic nerve activity, decreased from 0.58 before exercise to 0.36 after exercise, nonetheless without statistical significance.

Third, the healthy volunteers gained the benefits of decreasing systolic blood pressure, BMI, serum glucose and cholesterol, and physical fitness about one-minute bent-knee sit-ups, the sit and reach test, and the 3-minute gradual step test, all with statistical significance.

Fourth, Tai Chi synergy T1 exercise helped the practitioners reconstitute their immunity, with increased CD19+ B cells and CD3+ T cells, specifically in the subgroups of CD16+CD56+ NK/T cells, and decreased CD16-CD56- cytotoxic T cells.

As Tai Chi synergy T1 exercise is derived not only from Tai Chi exercise but also from Eight Trigrams Palms, form and will boxing, mantis boxing, Qigong, and Yoga, it belongs to a category of low-impact exercise with average METs = 3.69 ± 0.87, comparable to Tai Chi Chuan with approximately 6 METs, as a moderate aerobic exercise of 50-60% VO2 max [10, 11].

Moreover, the heart rate in most of the sessions of Tai Chi synergy T1 exercise rarely exceeds 100 beats per minute, and the maximal heart rate is 130 beats per minute. The respiratory exchange ratio in most of the sessions also lies between 0.8 and 1.0, indicating Tai Chi synergy T1 exercise as a low-intensity aerobic exercise.

Our study demonstrated that Tai Chi synergy T1 exercise lowered sympathetic tone but paradoxically increased parasympathetic nerve activity, which is consistent with many other reports of Tai Chi exercise. Tai Chi exercise can increase parasympathetic but decrease sympathetic modulation, therefore lowering arterial blood pressure [6–10]. Based on the HRV examination, Tai Chi exercise also demonstrated the effect of shifting the balance of the autonomic nervous system from sympathetic tone to parasympathetic tone [12, 13]. Tai Chi exercise practitioners retained better autonomic balance and demonstrated higher modulation of parasympathetic activity [12].

In our study, Tai Chi T1 exercise also improved systolic blood pressure, along with the metabolic profile, including serum glucose and cholesterol in healthy individuals, suggesting that it would be a reasonable treatment strategy adjunct to or independent of pharmaceutical agents for patients with essential hypertension, diabetes mellitus, or metabolic syndrome [12].

We found that Tai Chi T1 exercise had some effect on the number of innate and adaptive immunity-related cells. It has been long recognized that behavioral interventions may elicit immunity modifications, although this remains inconclusive until more powerful evidence arrives [14]. Serum level of IgG was significantly increased in men, and that of IgM was significantly decreased after practicing Tai Chi exercise for 2 months [15]. Another study found that Tai Chi exercise increases varicella-zoster virus specific cell-mediated immunity [16]. In addition, Chang et al. reported that the evidence-based results of Tai Chi exercise had small-to-moderate effects on body functions and structures, activities, and participation of physical component [17]. It is of note that our findings that Tai Chi synergy T1 exercise increased CD19+ B cells and CD16+CD56+ NK/T cells but decreased CD16-CD56- cytotoxic T cells are consistent with all the above-mentioned studies. The main function of CD19 on the surface of B cells is signaling transductions through B cell receptor-dependent or independent pathways to maintain humoral immunity, antigen-induced adaptive immunity, and induction of tolerance [18]. CD16+ CD56+ NK/T cells are important in their role of providing innate immunity by killing tumor and virus-associated cells [19]. To summarize, Tai Chi synergy T1 exercise enhanced innate and adaptive immunity through undetermined mechanisms.

Tai Chi synergy T1 exercise significantly improved physical fitness of the healthy volunteers in this study. Moreover, Tai Chi exercise may also prevent cardiovascular disease and have beneficial effects on cardiovascular disease risk factors for adults [20]. In a randomized study of 72 women with osteoarthritis, a 12-week Tai Chi exercise program was shown to reduce the pain and stiffness of joints, enhance the ability to perform daily activities, and strengthen abdominal muscles and balance [21]. In addition, balance intervention with Tai Chi exercise was repeatedly reported to improve balance control and prevent falls among elderly adults in several studies [22–24]. Other benefits of Tai Chi exercise include reduction of systolic blood pressure and diastolic blood pressure [25] and improved quality of life, mood, stress, activity tolerance, and cardiovascular function [26].

There are several limitations inherent to our study. Participants were recruited on willingness. Besides, the more participants involved, the larger practicing space required. Although the case-only design is based on a study sample consisting of cases only, it is an efficient approach to test for association between Tai Chi synergy T1 exercise and autonomic function, metabolism, and physical fitness of healthy individuals, when there is a reason to believe that independence assumptions were followed. However, the study demonstrated significant changes in autonomic control, metabolic profile, and physical function. In addition, while the numbers of adaptive immunity-related B cells and T cells were significantly altered, we failed to show the mechanism behind these changes and the specific subgroups of immune regulator cells could not be defined. Besides, the duration of practicing Tai Chi synergy T1 exercise was 10 weeks, and longer follow-up would be required to determine the 10-week effect of Tai Chi synergy T1 exercise, since some effects of exercise, such as improvement of cardiopulmonary function or bone density, might develop over a longer period.

In conclusion, our study demonstrated that Tai Chi synergy T1 exercise shifted the balance of autonomic control by increasing parasympathetic modulation and decreasing sympathetic nerve activity, decreased serum glucose, cholesterol, BMI, and systolic blood pressure, enhanced innate and adaptive immunity, and improved physical fitness and physical strength for healthy individuals practicing it for 10 weeks.

## Figures and Tables

**Table 1:** The details of 16 sessions of Tai Chi synergy T1 exercise.

Session	Duration	Heart rate	Respiratory exchange ratio	Energy consumption, Kcal
	(h:m:s)	(Mean ± SD)	(Mean ± SD)	(Mean ± SD)
1	0:04:16	87.71±4.90	0.81±0.08	7.71±1.37
2	0:08:52	87.65±5.53	0.87±0.08	22.68±3.74
3	0:13:24	90.12±6.09	0.85±0.07	36.05±5.99
4	0:17:06	89.47±6.37	0.94±0.08	48.27±7.40
5	0:22:30	92.41±6.97	0.83±0.06	77.51±10.21
6	0:27:54	110.82±16.72	0.88±0.07	100.49±13.19
7	0:30:53	114±14.86	0.94±0.06	113.51±14.72
8	0:34:17	117±13.45	0.85±0.09	133.49±17.59
9	0:37:25	130.24±13.40	0.87±0.06	152.45±18.95
10	0:40:58	106.71±7.45	0.85±0.06	171.00±20.65
11	0:44:28	109.06±8.74	0.88±0.08	188.12±22.06
12	0:48:31	100.12±13.20	0.84±0.1	206.82±24.49
13	0:53:44	124±9.88	1.00±0.05	224.4±22.45
14	0:55:18	88±10.65	0.72±0.06	229.25±19.64
15	0:59:17	96±9.34	0.85±0.09	238.16±24.25
16	0:63:20	94±12.22	1.01±0.09	245.29±25.22

*SD, *standard deviation.

**Table 2:** The effect of Tai Chi synergy exercise and regular walking on heart rate variability.

Tai Chi synergy exercise	Before	After	
	Mean (SD)	Mean (SD)	*p*
VLF [ln (ms2)]	5.93 (1.14)	6.52 (0.86)	<0.001*∗*
HF [ln (ms2)]	4.53 (0.87)	5.14 (1.04)	<0.01*∗*
LF [ln (ms2)]	5.11 (1.06)	5.50 (1.06)	<0.05*∗*
LF% [nu]	53.74 (18.18)	47.52 (20.16)	<0.05*∗*
HF% [nu]	30.58 (12.79)	34.89 (15.37)	<0.05*∗*
LF/HF [ln (ratio)]	0.58 (0.85)	0.36 (1.06)	NS
TP	6.66 (0.97)	7.19 (0.78)	<0.01*∗*

Regular walking	Before	After	
	Mean (SD)	Mean (SD)	*p*

VLF [ln (ms2)]	5.88 (0.82)	5.85 (0.81)	0.870
HF [ln (ms2)]	5.00 (0.72)	4.71 (0.93)	0.159
LF [ln (ms2)]	5.33 (0.82)	5.07 (0.81)	0.067
LF% [nu]	49.50 (17.85)	47.36 (15.42)	0.683
HF% [nu]	35.47 (13.92)	34.12 (14.23)	0.763
LF/HF [ln (ratio)]	0.34 (0.81)	0.37 (0.79)	0.901
TP	6.74 (0.67)	6.64 (0.65)	0.050

*∗p* < 0.05; *HF*, high frequency; *LF,* low frequency; *SD, *standard deviation; *TP*, total power; *VLF, *very low frequency.

**Table 3:** The effect of Tai Chi synergy exercise and regular walking on metabolism, bone density, and physical fitness.

Tai Chi synergy exercise	*n*	Before	After	
		Mean(SD)	Mean(SD)	*p*
SBP (mmHg)	30	130.37(14.72)	120.87(15.78)	0.001*∗*
DBP (mmHg)	30	77.70(9.56)	76.50(9.18)	0.376
BMI	27	24.59(3.58)	23.90(3.52)	<0.001*∗*
Glucose (mg/dl)	28	82.18(11.16)	72.86(7.89)	0.001*∗*
Cholesterol (mg/dl)	28	205.14(34.71)	195.82(31.62)	0.048*∗*
Bone density	17	0.56(1.41)	0.81(1.24)	0.138
One-minute bent-knee sit-ups (times)	18	16.44(7.40)	22.89(8.22)	<0.001*∗*
Sit and reach test (cm)	19	29.47(8.98)	34.79(7.67)	<0.001*∗*
Three-minute gradual step test (times)	24	57.39(7.71)	62.81(8.71)	0.005*∗*

Regular walking	n	Before	After	
		Mean(SD)	Mean(SD)	*p*

SBP (mmHg)	23	128.87(12.23)	128.70(11.97)	0.817
DBP (mmHg)	23	80.43(7.67)	79.65(7.79)	0.237
BMI	23	22.47(2.65)	22.68(2.92)	0.036*∗*
Glucose (mg/dl)	23	85.87(6.20)	85.65(6.59)	0.575
Cholesterol (mg/dl)	23	209.22(19.60)	209.26(19.32)	0.932
Bone density	23	0.59(1.03)	0.60(0.95)	0.846
One-minute bent-knee sit-ups (times)	23	16.78(4.35)	16.91(4.94)	0.479
Sit and reach test (cm)	23	29.78(4.57)	29.39(4.69)	0.025*∗*
Three-minute gradual step test (times)	23	55.96(3.50)	56.61(5.03)	0.126

*∗p* < 0.05; *BMI*, body mass index; *DBP, *diastolic blood pressure;* SBP*, systolic blood pressure; *SD, *standard deviation.

**Table 4:** The effect of Tai Chi synergy exercise on immune regulators.

	n	Before (cells/mm^3^)	After(cells/mm^3^)	
		Mean (SD)	Mean (SD)	*p*
CD16+CD56+ NK/T cells	20	9.81 (3.89)	12.99 (6.34)	0.037*∗*
CD16-CD56- cytotoxic T cell	20	49.81 (11.39)	43.72 (13.32)	0.035*∗*
CD19+ B cell	20	5.78 (2.15)	10.67 (3.77)	<0.001*∗*
CD3+ T cell	20	1114.65 (471.61)	2835.65 (1210.30)	<0.001*∗*
T cell	20	2654.17 (1058.31)	3898.12 (1910.57)	0.004*∗*

*∗p* < 0.05; *NK, *natural killer; *SD, *standard deviation.

## Data Availability

The data used to support the findings of this study are available from the corresponding author upon request.

## Abbreviations

BMI: Body mass index
ECGs: Electrocardiograms
FFT: Fast Fourier Transform
HRV: Heart rate variability
HF: High frequency
LF: Low frequency
MET: Metabolic equivalent of task
PSD: Power spectral density
RRI: R-R interval
TP: Total power.
